# Comparative external validation of ICH prognostic scores for discharge outcomes in the RES-Q multinational quality registry

**DOI:** 10.1093/esj/aakag105

**Published:** 2026-09-10

**Authors:** Ana Cláudia de Souza, Geraldo Neto, Nguyen Huy Thang, Nijasri C Suwanwela, Adin Nulkhasanah, Maria Epifania Collantes, Sheila Ouriques Martins, Sergii Moskovko, Mario A Munoz-Collazos, Louis Kroon, Jeyaraj Pandian, Adam Kobayashi, Zuzana Gdovinová, Natalia Ciobanu, Francisco Moniche, Cristina Tiu, Rupal Sedani, Robert Mikulík, P N Sylaja

**Affiliations:** Department of Neurology and Neurosurgery, Hospital Moinhos de Vento, Porto Alegre, Brazil; Stroke Research Program, International Clinical Research Center, St Anne’s University Hospital, Brno, Czech Republic; The People’s 115 Hospital Pham Ngoc Thach University of Medicine, Ho Chi Minh, Vietnam; Division of Neurology, Department of Medicine, Faculty of Medicine, Chulalongkorn University and Chulalongkorn Stroke Center, King Chulalongkorn Memorial Hospital, Bangkok, Thailand; Clinical Research Unit, National Brain Center Hospital Mahar Mardjono Jakarta, Jakarta, Indonesia; University of the Philippines Manila College of Medicine, Philippine General Hospital, Manila, Philippines; Department of Neurology and Neurosurgery, Hospital Moinhos de Vento, Porto Alegre, Brazil; Department of Neurology, Hospital de Clínicas de Porto Alegre, Universidade Federal do Rio Grande do Sul, Porto Alegre, Brazil; Neurology Department, Vinnitsa National Pirogov Memorial Medical University, Vinnitsa, Ukraine; Department of Neurology, Marly Clinic, Bogotá, Colombia; Department of Neurology, Steve Biko Academic Hospital, University of Pretoria, South Africa; Department of Neurology, Christian Medical College, Ludhiana, Punjab 141008, India; Department of Neurology, Stroke and Vascular Diseases Institute of Medical Sciences Faculty of Medicine—Collegium Medicum Cardinal Stefan Wyszynski University in Warsaw, Warsaw, Poland; Department of Neurology, P. J. Safarik University Kosice, Faculty of Medicine and University Hospital L. Pasteur, Kosice, Slovak Republic; Institute of Emergency Medicine, University of Medicine and Pharmacy Nicolae Testemitanu, Chisinau, Republic of Moldova; Department of Neurology, Stroke Unit, Instituto de Biomedicina de Sevilla (IBiS), Hospital Universitario Virgen Del Rocío/CSIC/Universidad de Sevilla, Seville, Spain; “Carol Davila” University of Medicine and Pharmacy, Bucharest, Romania; University Hospital Bucharest, Bucharest, Romania; World Stroke Organization, Geneva, Switzerland and Health Management Institute, Brno, Czech Republic; Health Management Institute, Brno, Czech Republic; International Clinical Research Centre, St. Anne's University Hospital, Brno, Czech Republic; Comprehensive Stroke Care Program, Sree Chitra Tirunal Institute for Medical Sciences and Technology, Thiruvananthapuram, Kerala, India

**Keywords:** intracerebral haemorrhage, prognostic score, external validation, outcomes

## Abstract

**Background:**

Prognostic scores are routinely applied after spontaneous intracerebral haemorrhage (ICH) to inform clinical decision-making and stratify risk; however, direct comparative validation in contemporary, multinational real-world cohorts remains scarce.

**Aims:**

To compare the discriminative performance of the ICH Score, max-ICH and ICH Functional Outcome Score (ICH-FOS) for discharge outcomes after spontaneous intracerebral haemorrhage in a large contemporary multinational cohort from the International Registry of Stroke Care Quality (RES-Q).

**Methods:**

We analysed consecutive adults with spontaneous ICH recorded in RES-Q between September 2024 and April 2025. Discharge outcomes were defined according to the modified Rankin Scale (mRS): mortality (mRS = 6), unfavourable functional outcome (mRS ≥ 4) and composite poor outcome (mRS 5-6). Discrimination was assessed using receiver operating characteristic curves and area under the curves (AUCs) with 95% confidence intervals. Areas under the curve were compared in the same complete-case cohort using global tests and pairwise DeLong contrasts. Score distributions were characterised descriptively to explore differences in granularity.

**Results:**

The complete-case cohort comprised 8450 patients from 56 countries. Median age was 63 (20) years, and 62% were male. Median National Institutes of Health Stroke Scale was 11 (12), Glasgow Coma Scale 14 (4) and median haematoma volume 16 (27) mL. Supratentorial ICH occurred in 82.1%, intraventricular haemorrhage in 31.5% and pre-admission oral anticoagulant use in 4.5%. Discharge mortality was 15.1% and favourable outcome (mRS 0-3) 56.6%. Discrimination was good for mortality (AUC 0.82 [0.81-0.83] for ICH Score, 0.81 [0.79-0.82] for max-ICH, 0.84 [0.83-0.86] for ICH-FOS; global *P* < .0001). For unfavourable outcome (mRS ≥ 4), AUCs were 0.77 (0.76-0.78), 0.81 (0.81-0.82) and 0.84 (0.83-0.85), respectively (*P* < .0001). For composite poor outcome (mRS 5-6), AUCs were 0.83 (0.82-0.84), 0.84 (0.83-0.85) and 0.87 (0.86-0.88) (*P* < .0001).

**Conclusions:**

In this large, contemporary, multinational registry cohort, all 3 prognostic scores demonstrated acceptable-to-excellent discriminative ability for discharge outcomes following spontaneous ICH. ICH Functional Outcome Score consistently achieved the highest discriminative performance across all endpoints, whereas max-ICH provided a pragmatic and robust alternative based on routinely available clinical variables.

## Graphical abstract

**Graphical Abstract:**
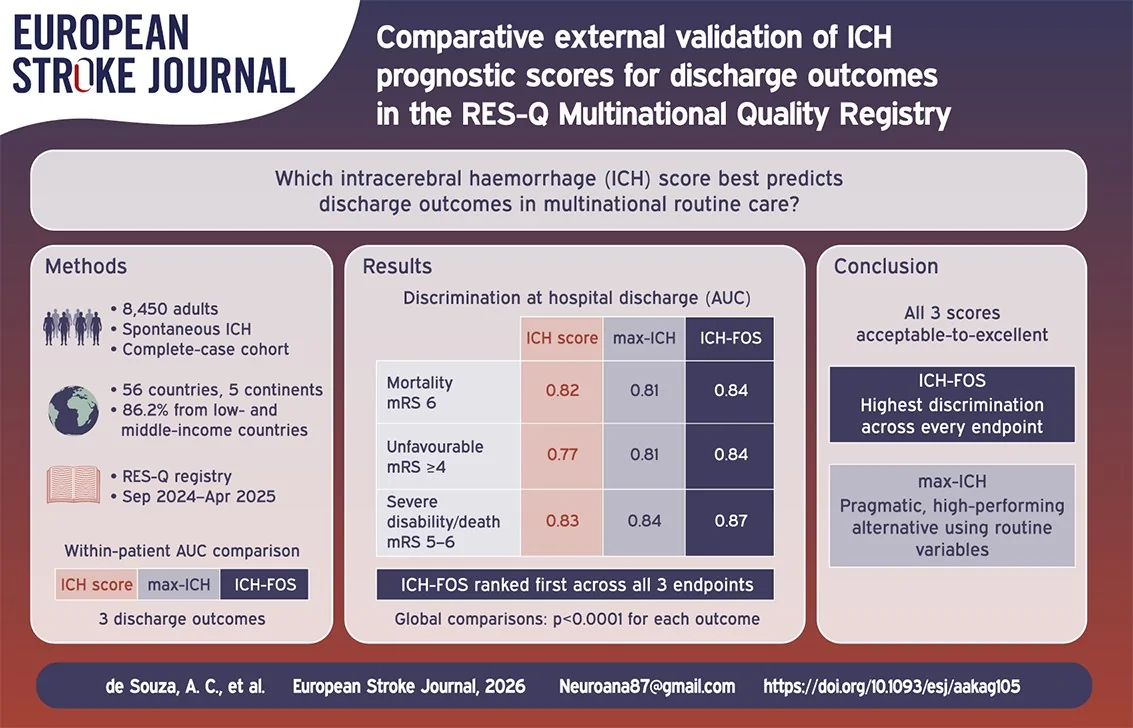

## Introduction

Spontaneous intracerebral haemorrhage (ICH) remains one of the most devastating stroke subtypes, with persistently high mortality and long-term disability.^1^ Although ICH accounts for a smaller proportion of strokes in high-income countries, its relative burden is disproportionately greater in low- and middle-income countries (LMICs), where health-system constraints and limited access to specialised neurocritical care may exacerbate poor outcomes.^1^^,^^2^ Despite its severity and substantial societal impact, therapeutic advances in acute ICH have historically lagged behind those achieved in ischemic stroke.^3^

Encouragingly, the acute ICH landscape is evolving. The INTEnsive care bundle with blood pressure Reduction in Acute Cerebral hemorrhage Trial (INTERACT3) trial demonstrated that implementing a goal-directed care bundle—addressing early intensive blood pressure lowering, fever treatment, glycemic control and rapid anticoagulation reversal—improved functional outcomes and reduced mortality, using measures broadly implementable across settings.^4^^,^^5^ Recent trials have further contributed to renewed momentum, including minimally invasive haematoma evacuation strategies and evidence for reversal of factor Xa inhibitor–associated ICH, albeit with safety trade-offs.^6^^,^^7^ In parallel, calls for more standardised, time-sensitive “Code ICH” pathways highlight the need for structured triage, monitoring and multidisciplinary decision-making.^8–10^

As care pathways become increasingly protocolised, accurate early prognostication becomes central to triage, intensity of monitoring, shared decision-making, benchmarking and risk stratification in pragmatic trials and quality-improvement programmes. Prognostic estimates may inform monitoring intensity, communication with patients and families, discharge planning, case-mix adjustment, benchmarking and risk stratification in clinical trials and quality-improvement programmes. Nevertheless, available prognostic scores differ substantially in their intended outcomes, predictor structure, complexity and clinical context, and should not be considered interchangeable.^11^

The original ICH Score remains the most widely recognised bedside grading scale and was developed primarily to estimate early mortality using a small number of readily available clinical and imaging variables.^12^ The max-ICH score represents a subsequent refinement developed in cohorts receiving maximal treatment, with the aim of improving prognostication while reducing the influence of early limitations of care.^13^^,^^14^ In contrast, the ICH Functional Outcome Score (ICH-FOS) was specifically developed to predict functional outcome and incorporates a broader range of clinical severity information.^15^ Together, these instruments represent 3 complementary approaches to ICH prognostication: a simple, mortality-oriented bedside score, a contemporary score derived from maximally treated patients and a more granular model focused on functional outcome.

The 3 scores were selected a priori according to 3 criteria: established clinical relevance, complementary prognostic purpose, model structure and the ability to calculate each score consistently using baseline variables systematically captured in the Registry of Stroke Care Quality (RES-Q). This approach also permitted direct, within-patient comparisons using the same complete-case cohort. Accordingly, the study was designed not as an exhaustive comparison of all published ICH prediction models, but as a focused evaluation of whether scores with different levels of complexity and different prognostic targets retain their expected performance when applied to a large, heterogeneous, multinational routine-care population.

Despite the widespread use of these instruments, direct comparative validation in contemporary multinational cohorts remains limited, particularly using standardised outcomes collected through international quality registries. Using data from the International RES-Q, we therefore compared the discriminative performance of the ICH Score, max-ICH and ICH-FOS for mortality, unfavourable functional outcome and severe disability or death at hospital discharge following spontaneous ICH.

## Methods

### Study design and data source

We conducted a registry-based validation study using RES-Q, an international web-based quality-improvement platform that captures standardised performance and outcome indicators across participating hospitals (www.qualityregistry.org). The platform has been used in prior multinational and country-level analyses, supporting its role as a real-world registry for monitoring acute stroke care processes and outcomes.^16^ For this study, we analysed consecutive entries recorded between September 2024 and April 2025.

### Participants

We included adults (≥18 years) admitted with spontaneous ICH. In accordance with the RES-Q protocol, we excluded ICH secondary to trauma, neoplasm, vascular malformation or haemorrhagic transformation of ischaemic stroke.

### Baseline variables and prognostic scores

Baseline variables recorded at presentation included demographics, clinical severity—modified Rankin Scale (mRS), National Institutes of Health Stroke Scale (NIHSS) and Glasgow Coma Scale (GCS); and imaging features (haematoma volume, supratentorial vs infratentorial location and intraventricular haemorrhage [IVH]). Pre-admission use of oral anticoagulants (OACs) was recorded as a binary variable. We evaluated 3 prognostic scores: the Intracerebral Haemorrhage Score (ICH Score), maximum Intracerebral Haemorrhage score (max-ICH) and Intracerebral Haemorrhage Functional Outcome Score (ICH-FOS). Table 1 summarises the main characteristics of the prognostic scores evaluated in the study.

**Table 1:** Overview of the prognostic scores used in the study.

Score	Main variables/components	Main prognostic focus	Practical note
**ICH Score**	GCS, age ≥80 years, haematoma volume, infratentorial location and IVH	Originally developed to estimate **30-day mortality** after spontaneous ICH	Simple and widely used; best for a quick bedside severity estimate.^12^
**max-ICH**	Admission NIHSS, age, IVH, oral anticoagulant use and haematoma volume thresholds according to lobar vs non-lobar location	Developed for **long-term functional outcome and mortality**, especially in maximally treated patients	More granular than the original ICH Score and explicitly includes anticoagulation.^13^
**ICH-FOS**	Age, admission NIHSS, GCS, blood glucose, ICH location, haematoma volume and intraventricular extension	Developed to predict **1-year functional outcome** after ICH	More outcome-oriented for disability/function, not just short-term mortality.^15^

Abbreviations: GCS = Glasgow Coma Scale; ICH = intracerebral haemorrhage; ICH-FOS = ICH Functional Outcome Score; IVH = intraventricular haemorrhage; NIHSS = National Institutes of Health Stroke Scale.

### Statistical analysis

Continuous baseline variables are summarised as mean (SD) and categorical variables as frequencies and proportions. Countries were classified as high-income countries (HICs) and LMICs according to the World Bank income classification. Outcomes at discharge were defined as: mortality (mRS = 6), unfavourable functional status (mRS ≥ 4) and composite poor outcome (mRS 5-6).

Area under the curves (AUCs) were compared within the complete-case cohort using nonparametric methods for correlated ROC curves. A global chi-square test was first used to assess equality of the 3 AUCs for each outcome. Pairwise DeLong contrasts were subsequently performed for ICH Score versus max-ICH, ICH Score versus ICH-FOS and max-ICH versus ICH-FOS, accounting for the covariance between ROC curves estimated in the same patients. Pairwise differences in AUC and corresponding two-sided *P* values were reported.^17^

To evaluate the potential for selection bias associated with the complete-case analysis, we compared the baseline characteristics of patients included in the primary analysis with those excluded due to incomplete score or discharge outcome data. Between-group differences were quantified using standardised differences rather than relying on significance tests, because conventional *P* values are highly sensitive to large sample sizes. For continuous and ordinal variables, the standardised difference was calculated as the difference between group means divided by the pooled standard deviation. For binary variables, the difference in proportions was standardised using the pooled binomial variance. Absolute standardised differences of < 0.10 were considered small, values from 0.10 to < 0.20 potentially meaningful, and values ≥0.20 indicative of more substantial imbalance. These thresholds were used as descriptive guides rather than formal hypothesis-testing criteria. Analyses were based on available observations for each variable; consequently, variable-specific denominators are reported. No imputation was performed for the primary analysis.

To explore whether differences in discrimination might be related to score granularity, we characterised the observed distribution of each prognostic score within the complete-case cohort. For each score, we reported the theoretical and observed ranges, number of observed categories, median and interquartile range, modal category and the proportions of patients classified within the most frequent 1 and 2 categories. Score distributions were displayed as discrete bar charts, with percentages calculated using the complete-case cohort as the denominator. These analyses were descriptive and were not intended to establish the independent contribution of score granularity or individual predictor components to differences in discrimination. All analyses were performed in Stata MP/14.0 for Windows (StataCorp, College Station, TX, USA).

**Ethics:** The study was approved by the Ethics Committee of St. Anne’s University Hospital in Brno (EK-FNUSA-04/2024), with patient consent waived due to data anonymization, ensuring compliance with international standards for patient safety and data security.

## Results

### Study population

During the study period, RES-Q included 26,357 adult admissions with spontaneous ICH that met eligibility criteria. Discharge mRS was documented in 22,554 patients. For paired (within-patient) comparisons of discrimination across all 3 prognostic scores, the primary analysis was restricted to the overlap complete-case cohort, which required non-missing values to compute ICH Score, max-ICH and ICH-FOS, and had documented discharge mRS (*n* = 8450). The complete-case cohort comprised patients from 56 countries across 5 continents. The cohort comprised 1165 (13.8%) patients from HICs and 7285 (86.2%) from LMICs.

Compared with patients excluded from the complete-case analysis, included patients were similar in age, sex, haematoma volume, IVH and previous OAC use, with absolute standardised differences below 0.10. The included cohort had lower prestroke disability (median mRS 0 [IQR 0-2] versus 1 [IQR 0-4]; standardised difference, 0.44), a slightly higher GCS distribution (median 14 [IQR 11-15] versus 14 [IQR 10-15]; standardised difference, 0.13) and a lower proportion of infratentorial ICH (17.9% versus 24.5%; standardised difference, 0.16). The difference in admission NIHSS was small (median 11 [IQR 6-18] versus 10 [IQR 5-17]; standardised difference, 0.09). Variable-specific denominators and complete comparisons are presented in Table 2. Characteristics of patients included in and excluded from the primary complete-case analysis.

**Table 2:** Characteristics of patients included in and excluded from the primary complete-case analysis.

Characteristic	Included, *n* = 8450	Excluded, *n* = 17,907	Standardised difference
**Age, years, median (IQR)**	63 (53-73)	63 (53-74)	0.022
**Male sex, *n*/*N* (%)**	5201/8449 (61.6)	10,641/17,907 (59.4)	0.044
**Prestroke mRS, median (IQR)**	0 (0–2), *n* = 8184	1 (0–4), *n* = 13,197	0.437
**NIHSS, median (IQR)**	11 (6–18), *n* = 8450	10 (5–17), *n* = 12,142	0.094
**GCS, median (IQR)**	14 (11–15), *n* = 8450	14 (10–15), *n* = 10,676	0.125
**Haematoma volume, mL, median (IQR)**	16 (8-35), *n* = 8450	18 (8-38), *n* = 6390	0.049
**Infratentorial ICH, *n*/*N* (%)**	1511/8450 (17.9)	3391/13,823 (24.5)	0.163
**IVH, *n*/*N* (%)**	2663/8450 (31.5)	4149/13,824 (30.0)	0.033
**Previous OAC use, *n*/*N* (%)**	344/7580 (4.5)	719/11,859 (6.1)	0.068

Abbreviations: GCS = Glasgow Coma Scale; ICH = intracerebral haemorrhage; IQR = interquartile range; IVH = intraventricular haemorrhage; mRS = modified Rankin Scale; NIHSS = National Institutes of Health Stroke Scale; OAC = oral anticoagulant.

### Discrimination performance for discharge outcomes

Table 3 summarises the performance of the prognostic scores evaluated. Across all 3 discharge outcomes, the ICH Score, max-ICH and ICH-FOS demonstrated acceptable and good to excellent discriminative performance, with ICH-FOS consistently outperforming the other scores and max-ICH generally performing better than the original ICH Score for functional outcome.

**Table 3:** Discrimination performance of ICH prognostic scores for discharge outcomes (*n* = 8450).

Outcome	ICH Score AUC (95% CI)	Max-ICH AUC (95% CI)	ICH-FOS AUC (95% CI)	Pairwise comparison	ΔAUC	Pairwise *P* value
**Discharge mortality (mRS = 6)**	0.82 (0.81-0.83)	0.81 (0.79-0.82)	0.84 (0.83-0.86)	ICH-FOS vs ICH Score	+0.0241	*P* < .0001
				ICH-FOS vs max-ICH	+0.0388	*P* < .0001
				ICH Score vs max-ICH	+0.0148	*P* = .0029
**Unfavourable functional outcome (mRS > =4)**	0.77 (0.76-0.78)	0.81 (0.81-0.82)	0.84 (0.83-0.85)	ICH-FOS vs ICH Score	+0.0656	*P* < .0001
				ICH-FOS vs max-ICH	+0.0253	*P* < .0001
				max-ICH vs ICH Score	+0.0403	*P* < .0001
**Composite poor outcome (mRS 5-6)**	0.83 (0.82-0.84)	0.84 (0.83-0.85)	0.87 (0.86-0.88)	ICH-FOS vs ICH Score	+0.0360	*P* < .0001
				ICH-FOS vs max-ICH	+0.0285	*P* < .0001
				max-ICH vs ICH Score	+0.0075	*P* = .0495

Abbreviations: AUC = area under the receiver operating characteristic curve; CI = confidence interval; ICH-FOS = Intracerebral Haemorrhage Functional Outcome Score; mRS = modified Rankin Scale. Global *P*-values compare discrimination across the 3 prognostic scores for each outcome. Pairwise comparisons are presented as Delta AUC for the first score listed minus the second score listed; positive Delta AUC values indicate higher discrimination for the first score listed. Pairwise *P* values are unadjusted; borderline results, including *P* values close to .05 (eg, *P* = .0495), should be interpreted cautiously.

For discharge mortality, the ICH Score showed slightly higher discrimination than max-ICH (Delta AUC, 0.0148; *P* = .0029), whereas ICH-FOS outperformed both the ICH Score (Delta AUC, 0.0241; *P* < .0001) and max-ICH (Delta AUC, 0.0388; *P* < .0001).

For unfavourable functional outcome, all pairwise differences were statistically significant. Max-ICH demonstrated higher discrimination than the ICH Score (Delta AUC, 0.0403; *P* < .0001), while ICH-FOS outperformed both the ICH Score (Delta AUC, 0.0656; *P* < .0001) and max-ICH (Delta AUC, 0.0253; *P* < .0001).

For the composite poor outcome, ICH-FOS demonstrated higher discrimination than the ICH Score (Delta AUC, 0.0360; *P* < .0001) and max-ICH (Delta AUC, 0.0285; *P* < .0001). The difference between max-ICH and the ICH Score was small and of borderline statistical significance (Delta AUC, 0.0075; *P* = .0495).

### Distribution and granularity of the prognostic scores

All theoretically possible categories were represented for each prognostic score in the complete-case cohort. The median ICH Score was 1 (IQR 0-2), the median max-ICH score was 2 (IQR 1-4) and the median ICH-FOS was 5 (IQR 3-8). The original ICH Score showed the greatest concentration within a limited number of lower categories: 31.1% of patients were classified as score 0, and 61.0% were classified within its 2 most frequent categories. The corresponding proportions within the 2 most frequent categories were 36.9% for max-ICH and 23.9% for ICH-FOS. Thus, max-ICH and particularly ICH-FOS showed broader distributions across their available ranges, whereas the ICH Score showed greater clustering within lower score categories (Figure 1).

**Figure 1:**
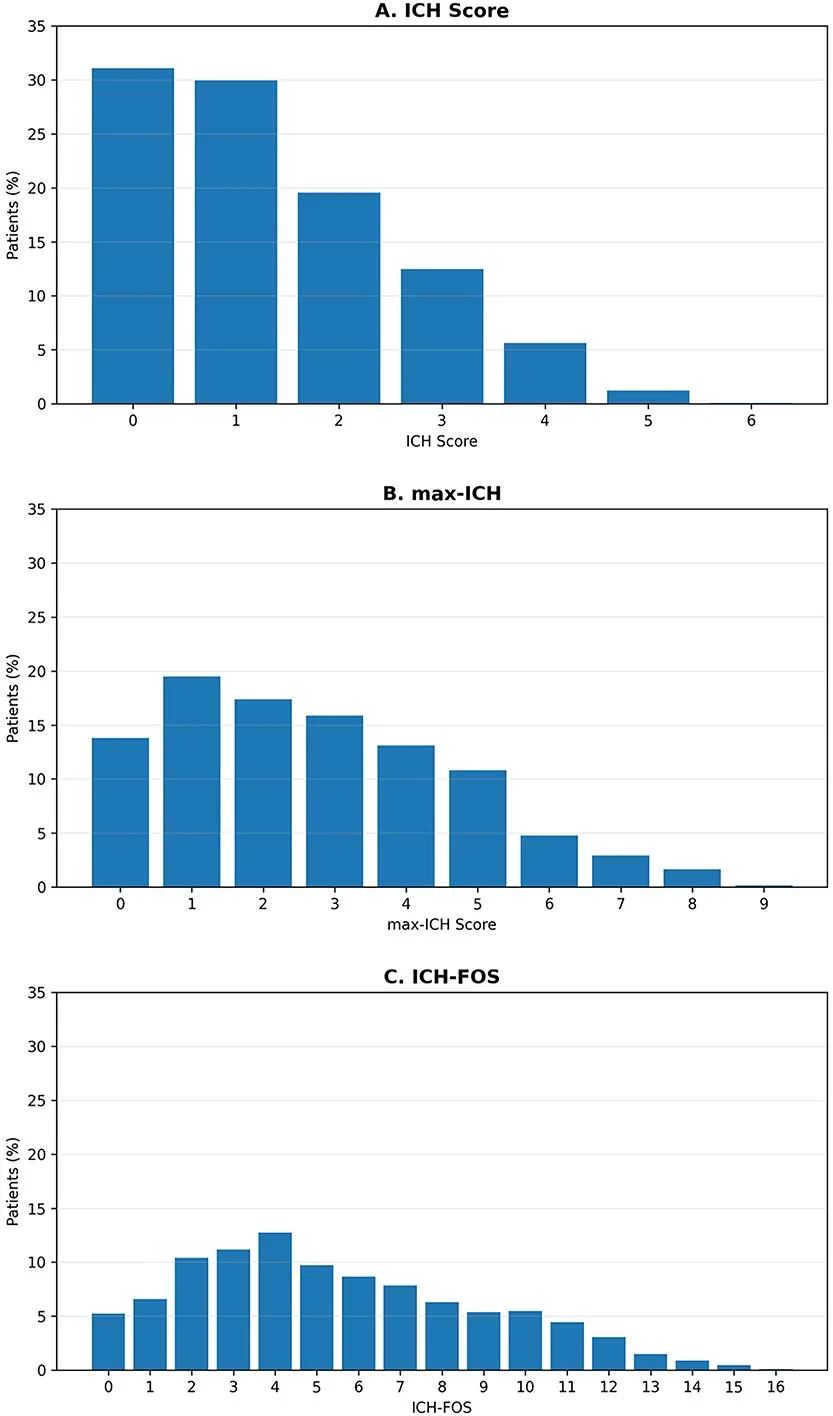
Distribution of the ICH Score, max-ICH and ICH-FOS in the complete-case cohort (*n* = 8450). Bars represent the percentage of patients within each score category. All theoretically possible categories were observed for each score. The original ICH Score showed greater concentration within lower score categories, whereas max-ICH and particularly ICH-FOS demonstrated broader distributions across their available ranges. Abbreviations: ICH = intracerebral haemorrhage; ICH-FOS = ICH Functional Outcome Score.

## Discussion

In this large, contemporary, multinational cohort from the RES-Q, the ICH Score, max-ICH and ICH-FOS demonstrated acceptable-to-excellent discrimination for early discharge outcomes after spontaneous ICH. Three findings are particularly relevant. First, discrimination for discharge mortality was uniformly good across scores, supporting the ongoing prognostic utility of classical severity markers (consciousness level, haematoma volume, infratentorial location and IVH). Second, for functional outcomes, max-ICH and ICH-FOS provided clear incremental discrimination over the original ICH Score, suggesting that refinements intended to better model disability gradients translate into improved performance in routine-care populations. Third, ICH-FOS consistently outperformed both scores in all discharge endpoints. Given the predominance of LMIC settings, these results may be particularly informative for routine-care prognostication in resource-variable health systems.

The mortality AUCs observed in our cohort, ranging from 0.81 to 0.84, are consistent with previous external validations of the ICH Score and max-ICH.^13^^,^^14^^,^^18^ The superior discrimination of max-ICH over the ICH Score for unfavourable functional status (0.81 vs 0.77) aligns with its development objective to improve outcome prediction in maximally treated patients and mitigate biases related to early care limitations.^13^^,^^14^ This suggests that max-ICH more effectively captures gradients of functional dependence than the original ICH Score.

ICH Functional Outcome Score demonstrated the highest discrimination for both unfavourable outcome and the composite endpoint of severe disability or death (AUC up to 0.87), consistent with prior reports of mid-0.8 AUCs for disability-focused endpoints.^15^^,^^17^^,^^18^^,^^19^ Although ICH-FOS was originally developed to predict 1-year functional outcome, its strong performance at discharge is clinically plausible. Early and longer-term disability after ICH share major baseline determinants, including age, neurological severity, level of consciousness, haematoma volume, location, intraventricular extension and metabolic stress. Moreover, the original validation of ICH-FOS showed good discrimination not only at 1 year but also for earlier 30-day, 3-month and 6-month functional and mortality outcomes,^15^

The score-distribution analysis provides additional insight into the observed differences in discrimination. Although all theoretically possible categories were represented, the original ICH Score showed substantial clustering within a small number of lower categories, whereas max-ICH and, particularly, ICH-FOS distributed patients more broadly across their available ranges. A wider ordinal distribution may reduce ties and permit finer separation of patients with different baseline risks.

Granularity alone, however, is unlikely to account fully for the superior performance of ICH-FOS. The score also combines complementary neurological, metabolic and imaging domains, including both NIHSS and GCS, blood glucose and differently weighted clinical and radiological predictors. Its higher discrimination therefore likely reflects the combined effects of broader ordinal resolution, predictor selection and weighting. These findings illustrate an important clinical trade-off: simpler scores facilitate rapid bedside calculation and communication, whereas more detailed instruments may provide finer risk stratification when all required variables are available.

From a practical perspective, our results suggest that the choice of score should depend on the intended prognostic purpose. ICH Functional Outcome Score may be preferred when estimating the probability of meaningful functional dependence or severe outcome with maximal discrimination. Max-ICH represents a practical alternative when simplicity or variable availability limits implementation of more complex tools, while maintaining strong performance for disability endpoints. The original ICH Score remains useful for rapid bedside mortality estimation but appears comparatively less discriminative for functional outcomes in contemporary practice.

These findings should nevertheless be interpreted in the context of the outcome assessment used. Discharge mRS is clinically relevant for early prognostication, care planning, resource allocation and quality benchmarking, but it is not equivalent to long-term functional recovery. Neurological improvement may continue for several months after ICH, and discharge status may be influenced by length of stay, access to rehabilitation, interhospital transfer and country-specific discharge practices. The present results therefore describe discrimination for early hospital outcomes and should not be directly extrapolated to 90-day or longer-term prognosis.

Prognostic studies in intracerebral haemorrhage are also vulnerable to self-fulfilling prophecy, whereby early expectations of poor outcome may influence treatment intensity and decisions regarding limitation or withdrawal of life-sustaining therapies.^19^ The present findings should not be interpreted as endorsing deterministic use of prognostic scores for care limitation. All evaluated instruments provide population-level risk estimates rather than individualised predictions and do not account for dynamic clinical evolution, treatment response, in-hospital complications or patient-centred values. Goals-of-care decisions after ICH should therefore rely on serial clinical assessment, multidisciplinary deliberation and shared decision-making with patients and families, with prognostic scores serving as adjunctive tools. Their principal value lies in structured risk communication, case-mix adjustment for benchmarking and stratification in clinical research and quality-improvement initiatives.^3^

Although the influence of self-fulfilling prophecy cannot be entirely ruled out in observational registry data, an important strength of the present analysis is its multinational scope across diverse health-care systems. Practices regarding prognostic disclosure, intensity of care and withdrawal of life-sustaining treatment vary substantially across countries and regions, and no single prognostic score is uniformly embedded in decision-making worldwide. This heterogeneity reduces the likelihood that outcomes within the RES-Q cohort are systematically driven by reliance on one specific prognostic model, thereby mitigating—though not eliminating—the potential impact of self-fulfilling prophecy on comparative performance estimates.

This study has several strengths. It used a very large, contemporary, multinational dataset derived from a global stroke quality registry, enabling robust head-to-head comparison of 3 widely used prognostic scores within the same cohort. This design reduces between-study heterogeneity that complicates cross-paper comparisons and reflects real-world, protocolised ICH care across diverse health-system contexts. Registry of Stroke Care Quality captures prognostication under routine clinical conditions, including real-world treatment decisions, making these findings particularly relevant for benchmarking and quality-improvement applications rather than idealised trial settings.

Several limitations should be acknowledged. The comparative analyses were restricted to patients with complete data for all 3 scores and discharge mRS, which may have introduced selection bias. Compared with excluded patients, those included in the primary analysis had lower prestroke disability, slightly higher GCS values and a lower frequency of infratentorial ICH, although differences in age, sex, NIHSS, haematoma volume, IVH and previous oral anticoagulant use were small. The analytic cohort may therefore underrepresent patients with greater prestroke disability or infratentorial haemorrhage. Although the paired complete-case approach ensures that the scores are compared within the same individuals, it does not eliminate bias associated with non-random data completeness.

The outcomes were assessed at hospital discharge rather than at a standardised follow-up time point. Admission date and length of stay were unavailable, preventing adjustment for duration of hospitalisation or evaluation of differences in discharge timing across centres. Longer-term outcomes at 90 or 180 days would provide a more complete assessment of sustained functional recovery. The mRS assessment in routine clinical practice may be subject to inter-rater variability, potentially attenuating estimates of discrimination. Finally, we evaluated scores calculated from baseline variables and did not assess whether delayed or repeated prognostic assessment would improve performance. Models reassessed after the first hours or days may incorporate early neurological evolution, haematoma expansion, treatment response and in-hospital complications; however, RES-Q does not systematically collect repeated score components at standardised post-admission time points across participating centres.

## Conclusions

In a large contemporary multinational registry cohort, the ICH Score, max-ICH and ICH-FOS showed acceptable-to-excellent discrimination for discharge outcomes after spontaneous ICH. ICH Functional Outcome Score consistently provided the highest discrimination, while max-ICH offered a pragmatic high-performing alternative using routinely available variables.

## Data Availability

The data underlying this article were provided by the International Registry of Stroke Care Quality (RES-Q) under specific governance and data access agreements and therefore cannot be shared publicly. Requests for access to de-identified data may be considered on reasonable request, subject to approval by the RES-Q governance structure and applicable ethical and institutional requirements.

## Trial registration

Not applicable. This was an observational study based on the secondary analysis of data from a multinational quality-of-care registry and did not involve the prospective assignment of participants to a healthcare intervention.
